# *Micromonospora profundi* TRM 95458 converts glycerol to a new osmotic compound

**DOI:** 10.3389/fmicb.2023.1236906

**Published:** 2023-09-07

**Authors:** Di Lu, Hong-ling Shen, Lei Wang, Chuan-xing Wan

**Affiliations:** ^1^Key Laboratory of Protection and Utilization of Biological Resources in Tarim Basin Co-funded by Xinjiang Production & Construction Corps and The Ministry of Science & Technology, Tarim University, Alar, China; ^2^College of Life Sciences and Technology, Tarim University, Alar, China

**Keywords:** ABAGG, compatible solutes, conversion of glycerol, *Micromonospora profundi* TRM 95458, salt-tolerant rhizobacteria

## Abstract

Plant growth and agricultural productivity was greatly limited by soil salinity and alkalization. The application of salt-tolerant rhizobacteria could effectively improve plant tolerance to saline-alkali stress. *Micromonospora profundi* TRM 95458 was obtained from the rhizosphere of chickpea (*Cicer arietinum* L.) as a moderate salt-tolerant rhizobacteria. A new osmotic compound (ABAGG) was isolated from the fermentation broth of *M. profundi* TRM 95458. The chemical structure of the new compound was elucidated by analyzing nuclear magnetic resonance (NMR) and high-resolution mass (HRMS) data. *M. profundi* TRM 95458 could convert glycerol into ABAGG. The accumulation of ABAGG varied depending on the amount of glycerol and glycine added to the fermentation medium. In addition, the concentration of NaCl affected the ABAGG content obviously. The highest yield of ABAGG was observed when the salt content of the fermentation medium was 10 g/L. The study indicated that salt stress led to the accumulation of ABAGG using glycerol and glycine as substrates, suggesting ABAGG might aid in the survival and adaptation of the strain in saline-alkaline environments as a new osmotic compound.

## Introduction

Salinity is one of the major abiotic stress factors that threaten plant growth and agricultural productivity (Wang et al., 2022). This phenomenon is attributed to osmotic stress, which leads to water loss and impedes the absorption of water by plant roots. Additionally, ionic stress caused by high levels of Na^+^ and Cl^−^ ions can result in toxicity and hinder the uptake of other essential ions (Robles et al., 2018). Due to the widespread drought conditions in recent years and the lack of confident water resources, there has been an increase in saline soil levels, particularly in arid regions. Soil microbes such as plant growth-promoting rhizobacteria (PGPR) play a vital role in improving plant growth, soil health, abiotic stress and enhance crop productivity (Kong et al., 2018; Kumar Arora et al., 2020; Kumawat et al., 2020). The rhizosphere refers to the soil zone surrounding plant roots, which has a significant impact on the biological and chemical properties of the soil. In the rhizosphere, bacterial concentrations are approximately 10–1,000 times higher compared to the bulk soil (Gouda et al., 2018; Liu et al., 2020). The microorganisms present in their rhizosphere play a crucial role in enhancing the plants’ stress tolerance (Ferreira et al., 2019; Liu et al., 2022). The application of PGPR has effectively improved plant tolerance to saline-alkali stress (Nordstedt and Jones, 2020). Actinomycetes have been reported to alleviate salt stress. For instance, *Streptomyces* sp. strain PGPA39 isolated from agricultural soil has been stated to alleviate salt stress in tomato plants (Rani et al., 2020). Actinomycetes produce many compatible solutes such as ectoine, trehalose, glycine betaine, and glucosylglycerol, and thus have a great capacity to increase salt-tolerance (Wang et al., 2021; Xu et al., 2021; Narsing Rao et al., 2022). *Micromonospora* is a highly productive genus within the rare actinomycetes group, with over 740 bioactive microbial metabolites discovered up to date (Mercedes et al., 2018). Most of the *Micromonospora* species contain salt-tolerant gene clusters such as NAGGN gene cluster, which may be a resource for discovering new osmotic compound. In light of the tremendous potential of actinomycetes in mitigating salt stress conditions, this study focused on isolating and identifying the salt stress tolerance of actinomycetes in the rhizosphere soil of Chickpea (*Cicer arietinum* L.). Chickpea is a crucial pulse crop that is widely cultivated and consumed globally, with a particular emphasis on Afro-Asian countries (Varshney et al., 2021; Grasso et al., 2022). Chickpea is a popular food crop in China, particularly in the northern and southern Tianshan Mountains of Xinjiang. Mulei County in Xinjiang is known as the hometown of chickpeas and is the largest chickpea planting base of China. This crop is well-suited for the region due to its ability to withstand salt stress conditions. Soil salinisation is a global and dynamic problem which is predicted to intensify in the future under the changing realms of climate (Amro et al., 2022). Rhizobacteria including *Rhizobium* and *Micromonospora* are abundantly existed in the rhizosphere of chickpea. Previous studies have shown that plant growth-promoting rhizobacteria (PGPR), which are beneficial soil microorganisms, can enhance plant growth and yield even under stress conditions. The salt-tolerant antagonistic bacteria CZ-6 has the potential to act as a biological control agent in saline soil. It is expected that the addition of salt-tolerant antagonistic bacteria will help alleviate plant damage and economic losses caused by pathogenic fungi and salt stress (Zhou et al., 2021). Halophytes, which exhibit extreme salt tolerance, are cultivated in saline-alkaline environments. The microorganisms present in their rhizosphere play a crucial role in enhancing the plants’ stress tolerance (Ferreira et al., 2019). However, there are few studies on the salt-tolerant active monomer components of chickpea soil microorganisms. In order to find out the beneficial actinomyces and active ingredient with salt tolerance, *M. profundi* TRM 95458 was isolated and screened from the rhizosphere soil of chickpea in Mulei County, Xinjiang. The investigation of compatible solutes in *M. profundi* TRM 95458 resulted in findinig a new osmotic pressure regulator ABAGG.

## Materials and methods

### General experimental procedures

The high-performance liquid chromatography (LC-20AT, Shimadzu) with an Agilent ODS column (4.6 × 250 mm) was used for HPLC analysis. Preparative HPLC (pHPLC) was carried out using Waters 2,767 coupled with a Shim-park RP-C18 column (250 × 20 mm i.d., Shimadzu). NMR spectra were measured in MeOD at 298 K on a Bruker Ascend^™^ 500 M NMR (^1^H NMR, 500 MHz; ^13^C NMR, 125 MHz) spectrometer with TMS as internal standard. HRMS (ESI) m/z measurements were obtained on a Thermo Scientific Q Exactive mass spectrometer. SEM photos were taken by an S3400n scanning electron microscopy (Hitachi). Software MEGA 5.05 was used to establish phylogenetic tree.

### Isolation of *M. profundi* TRM 95458

The strain was isolated from soil samples taken from the chickpea rhizosphere in Mulei County, Xinjiang (90.14° East longitude, 43.56°North and 1,562 m altitude) in July, 2021. This was achieved using the dilution coating plate method with 1/10 ISP2 medium (0.4% yeast extract, 1% wort, 0.4% glucose, 0.1% NaCI, and 1.6% agar, which was made up to 1 L with water, pH 7.0) in a 28°C constant temperature culture. The strain TRM 95458 was further cultured by a glycerol-amino acid medium.

### Taxonomic identification of *M. profundi* TRM 95458

#### Morphological observation

Scanning electron microscopy required a solid plate culture, using a blade to cut off a block (size of about 0.5 cm × 0.5 cm). The excess medium was cut off, with the mycelium medium of thin and uniform thickness. The morphology of mycelium, the growth of aerial mycelium and basal mycelium, whether the mycelium produces spore filaments, and the arrangement and shape of spore filaments were observed and recorded.

### Molecular biological identification of strain TRM 95458

#### Extraction of genomic DNA from strain TRM 95458

The TRM 95458 cells on the culture plate were collected and placed in a sterile 1.5-mL centrifuge tube, and 480 μL 1 × TE buffer was added. Then, 20 μL of lysozyme (50 mg·mL^−1^) were added, and it was put in a 37°C water bath overnight. Each tube had 50 μL 20% SDS, 5 μL 20 mg·mL^−1^ protease K and was placed in a 60°C water bath for 2 h. Then, 55 μL of phenol: chloroform: isoamyl alcohol (25:24:1) were added, the tubes were centrifuged at 12,000 rpm for 5 min. The supernatant was moved into another centrifuge tube, which was repeated twice. The supernatant was mixed with 300 μL of 95% isopropanol and 70 μL of sodium acetate (3 mol·L^−1^), centrifuged at 12,000 rpm for 10 min, and the supernatant was discarded. The centrifuged product was washed with 500 μL of 70% ethanol once, centrifuged at 12000 rpm for 5 min, and the supernatant was discarded to volatilise the ethanol completely. The DNA at the bottom was fully dissolved with 30 μL of sterile ultra-pure water, and the quality of DNA extraction was detected by 1% agarose gel electrophoresis. The extracted DNA was stored in a refrigerator at −20°C.

### Amplification of 16S rRNA gene of strain TRM 95458

The 16S rRNA gene fragment in the genomic DNA of actinomycetes was amplified by using the universal primers 27F (5’-AGAGTTTGATCCTGGCTC-3′) and 1492R (5’-CGGCTACCTTGTTACGACTT-3′). The 50 μL PCR reaction system was as follows: 34 μL dd H_2_O, 5 μL 10× buffer, 2.5 μL dNTPs, 2 μL primer 27F (10 μmol·L^−1^), 2 μL primer 1492R (10 μmol·L^−1^), 2 μL 50% DMSO, 0.5 μL Taq DNA polymerase and 2 μL template DNA.

The PCR reaction conditions were pre-denaturation at 94°C for 4 min. Denaturation was done at 94°C for 1 min, annealing at 56°C for 1 min, extension at 72°C for 2 min for 30 cycles and then total extension at 72°C for 8 min. The reaction was detected by 1% agarose gel electrophoresis. The sequencing results were spliced by DNAMAN5.2, and the sequences were compared with the published strains in the GenBank database by BLAST. The 16S rRNA gene sequences of the published strains with high similarity were downloaded. The phylogenetic tree was constructed by MEGA5.05 software to determine the taxonomic status of strain TRM 95458.

### Genome sequencing of strain TRM 95458

Strain TRM 95458 was cultured in a liquid medium. When the strain grew to the logarithmic phase, the bacteria were collected and sent to the Xi’an Branch of Beijing Qingke Biotechnology Co., Ltd. for equencing by Nanopore PromehION.

### Fermentation, isolation, and purification of compounds

A single colony in the solid medium was selected to seed the liquid medium (0.4% yeast extract, 1% wort, 0.4% glucose, and 0.1% NaCI, which was made up to 1 L with water, pH 7.0), and incubated at 30°C on a rotary shaker (150 rpm) to produce fermentation for five days. Then, 4% of the seed liquid medium was inoculated into the fermentation medium (glycerol 2%, peptone 1%, glycine 1% and yeast extract 1%, which was made up to 1 L with water, pH 7.0) and cultured at 30°C for 7 days. The bacterial solution was centrifuged, and the fermentation products were separated into fermentation broth and mycelia. The fermentation broth was adsorbed with D101 macroporous resin and washed with distilled water to remove sugars, salts, and extracellular proteins. Then, it was eluted with 30% methanol. Finally, 95% methanol was used to elute into colourlessness, and the eluent was concentrated under reduced pressure by a rotary evaporator to obtain the crude extract of secondary metabolites. The crude extract products were packed into an ODS column (50 cm × 6.0 cm) and eluted with MeOH-H_2_O (30%, 50%, 80%, and 100% MeOH) to give four fractions. The 80% methanol eluent was further purified by preparative HPLC. The solvent systems for pHPLC were A: 30% MeOH-H_2_O (0.075% HCOOH), B: 100% MeOH and a flow rate of 10 mL/min. The gradient elution conditions were 0–40 min, 0–65% B; 40–45 min, 65–100% B; and 45–50 min, 100% B. The DAD detector wavelength was 350 nm. Compounds 1, 2, 3, and 4 were harvested from the peaks at 21.85 min, 19.38 min, 28.28 min, and 33.13 min, respectively.

### Functional determination of converting from glycerol to ABAGG by TRM 95458

The purified ABAGG was prepared in a 1.0 mg/mL mother liquor. It was then diluted to concentrations of 1.0, 0.5, 0.25, 0.125, 0.0625, 0.03125, and 0.0156 mg/mL to create a standard curve using the area normalisation method with HPLC. Varying amounts of glycerol and glycine were added to the fermentation media, the fermentation broth was subjected to detect the production amount of ABAGG.

### The relationship between salt stress and ABAGG accumulation in TRM 95458

The effect of different concentrations of NaCl (2.5, 5, 10, 15, and 20 g/L) was investigated on the production of ABAGG in TRM 95458 fermentation broth.

## Results

### Taxonomy of the isolated organism

**Figure 1:**
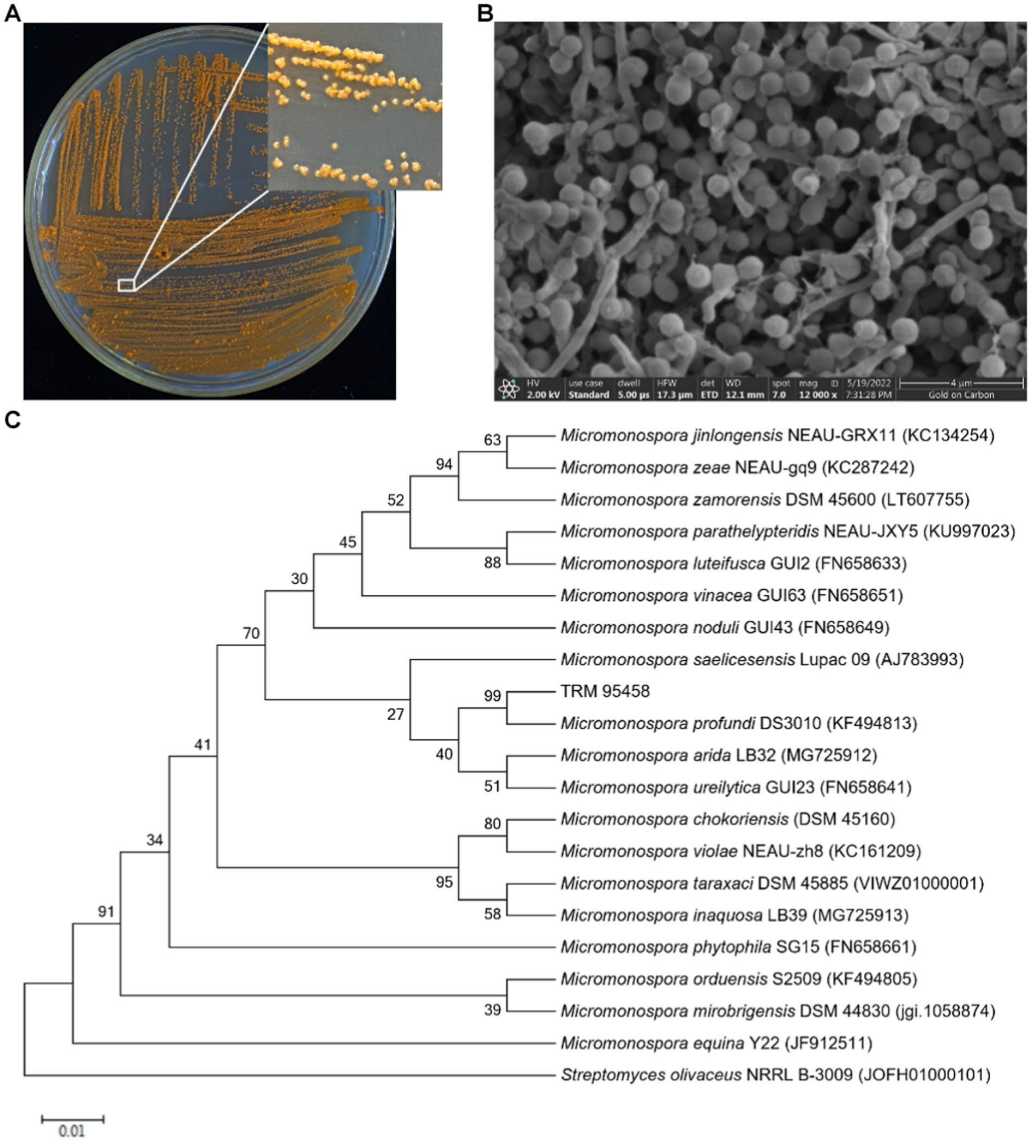
Colony characteristics and scanning electron micrograph of TRM95458 grown on ISP2 agar at 28°C for 7 days. **(A)** Colony characteristics of TRM95458. **(B)** The spores of TRM95458. **(C)** Neighbour-joining phylogenetic tree from the 16S rRNA sequences of TRM95458 and related species constructed by MEGA 5.05. Numbers at nodes indicate levels of bootstrap support (%) based on a neighbour-joining analysis of 1,000 resampled datasets; only values >50% are given. NCBI accession numbers are given. Bar, 0.01 nucleotide substitutions per site. *Streptomyces olivaceous* NRRL B-3009 was selected as the outgroup.

The strain TRM95458 isolated from chickpea rhizosphere soil was an actinomycetes judging by the single warty-globular spore of colony (Figure 1A) and the toruliform spores observed in the scanning electron microscopy (SEM) photo (Figure 1B). Based on 16S rRNA phylogenetic analysis, the strain was further classified as *M. profundi*, with 100% similarity to *M. profundi* DS3010 (Figure 1C).

### Structure elucidation of the new compound

**Figure 2:**
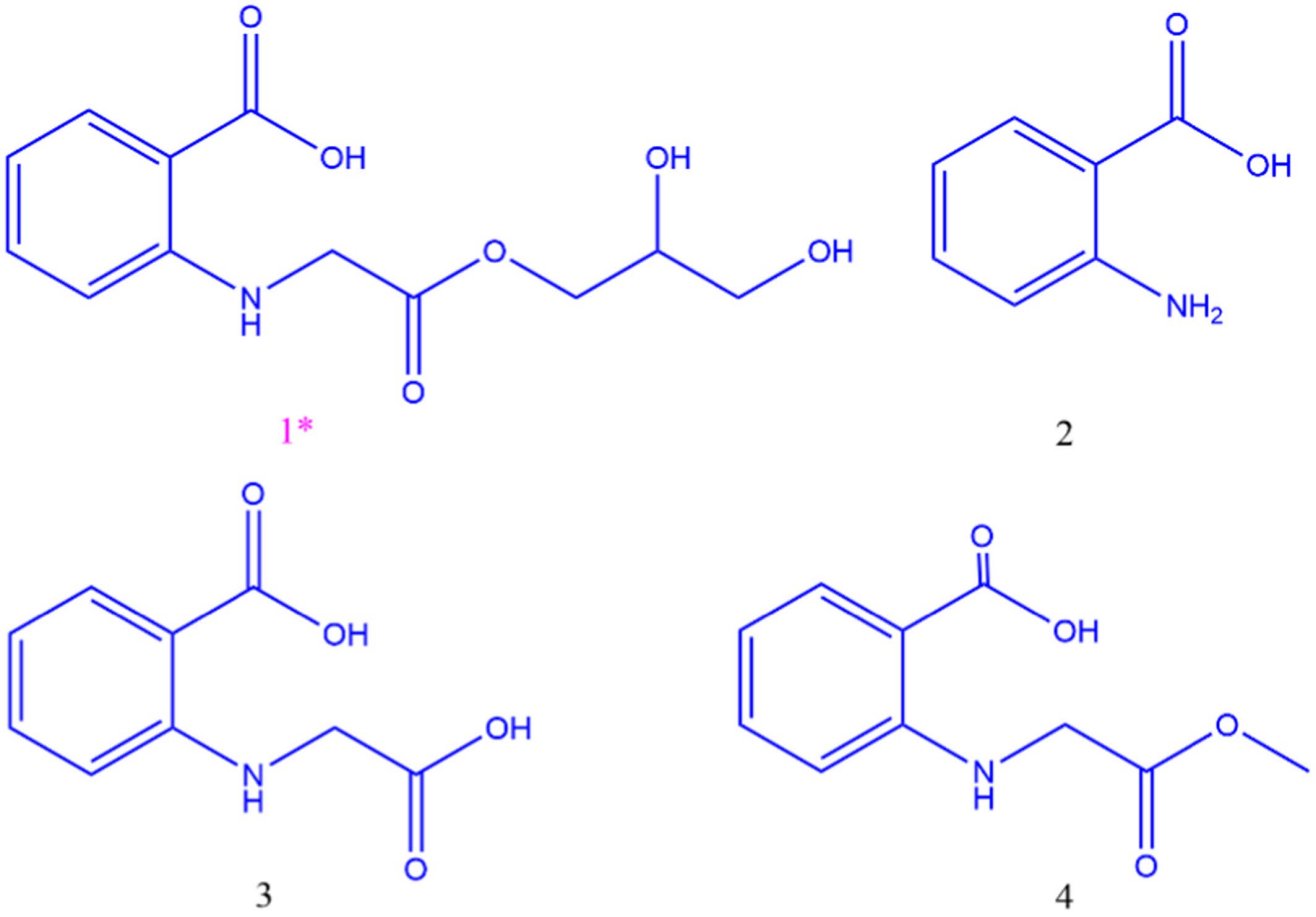
Structure of compounds 1, 2, 3,4.

**Figure 3:**
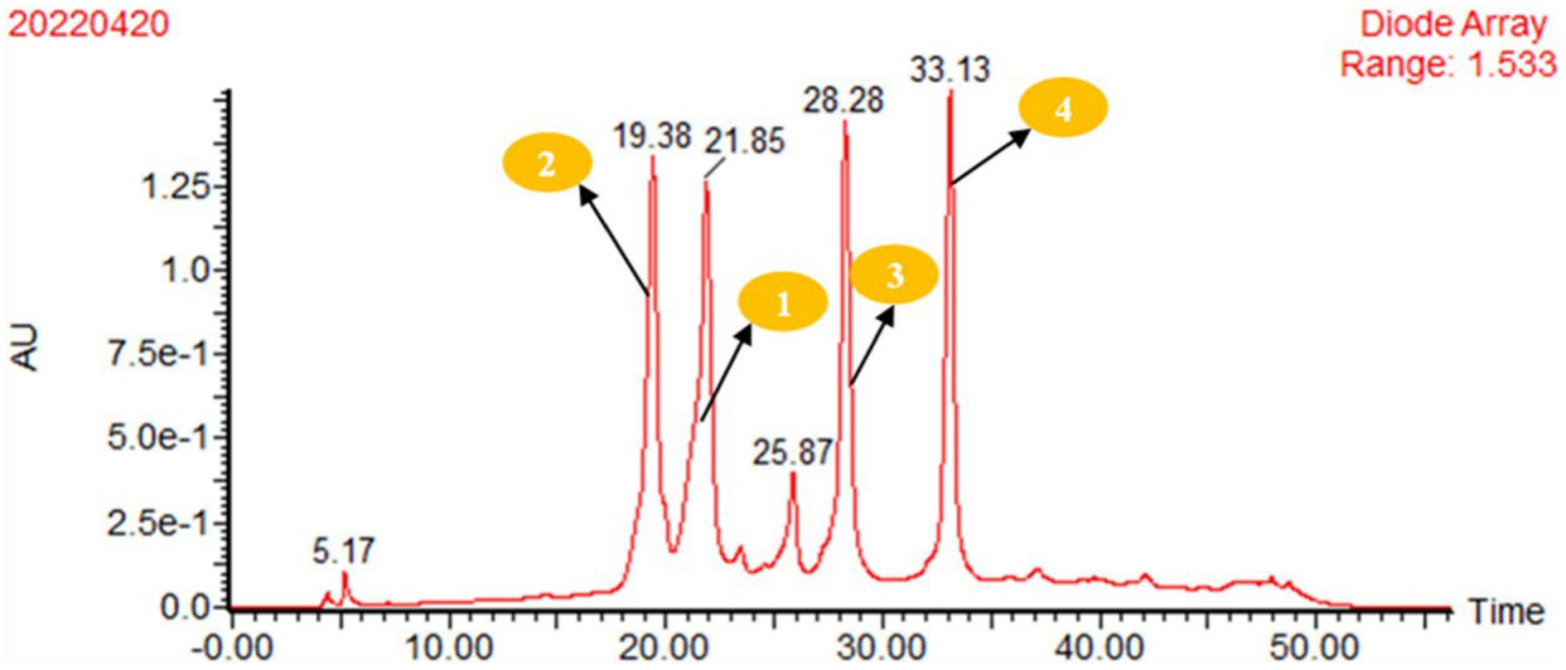
pHPLC analysis of the crude extract of strain TRM95458 fermented in liquid fermentation medium (Glycerol 2%, peptone 1%, glycine 1%, yeast extract 1%), 80% methanol eluent was purified by preparative HPLC, Conditions: A: 30% MeOH-H2O (0.075% HCOOH); B: 100% MeOH; flow rate:10mL/min; gradient elution conditions: 0–40 min 0–65% B; 40–45 min 65–100%B; 45–50 min 100%B; DAD detector wavelength: 350 nm. 2, tR = 19.38 min; 1, tR = 21.85 min; 3, tR = 28.28 min; and 4, tR = 33.13 min. The crude extract was isolated by pHPLC equipped.

**Figure 4:**
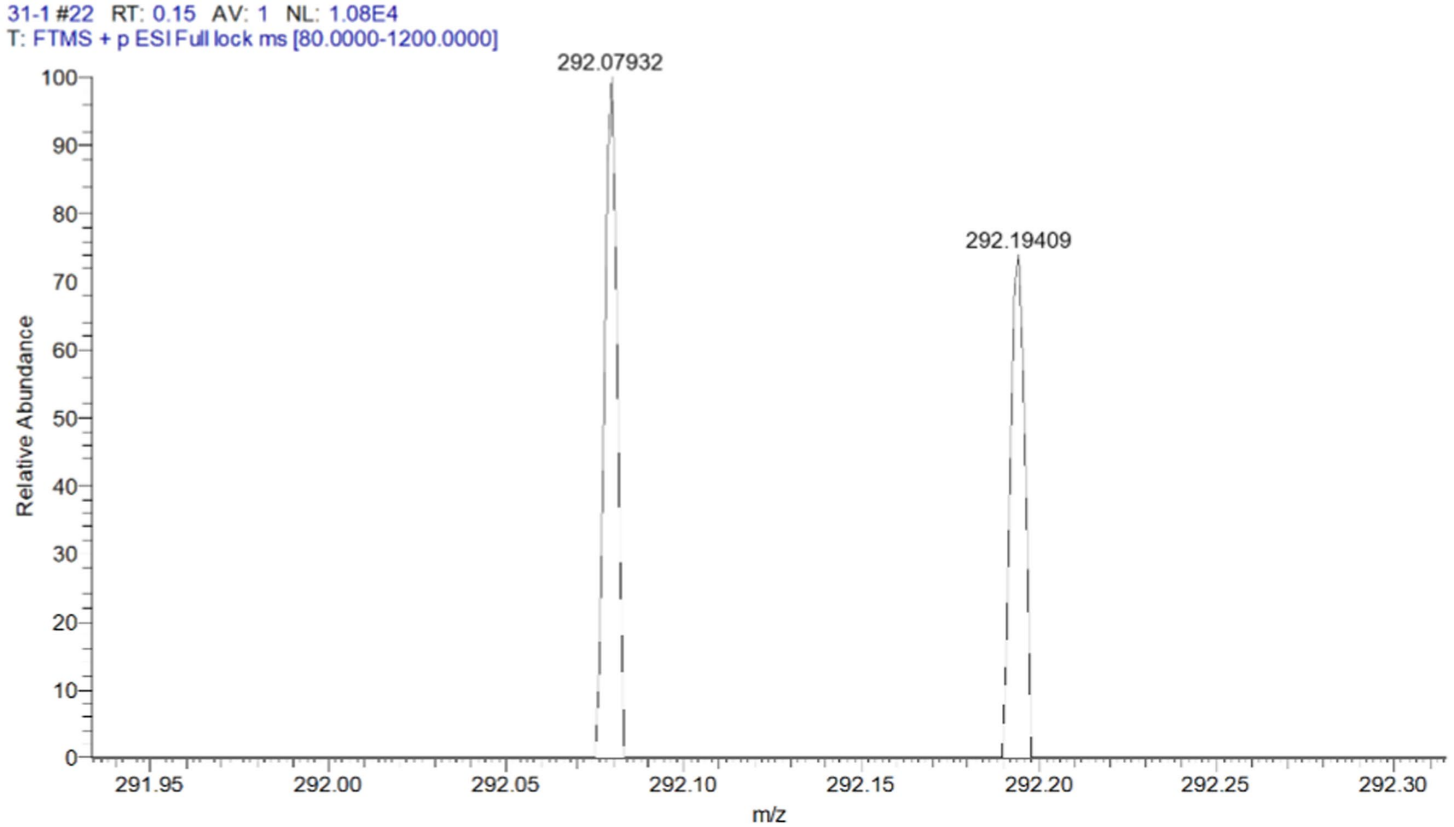
HRMS spectrum of compound 1 C12H15NaNO6+ (M+Na)+ 292.0791584.

**Figure 5:**
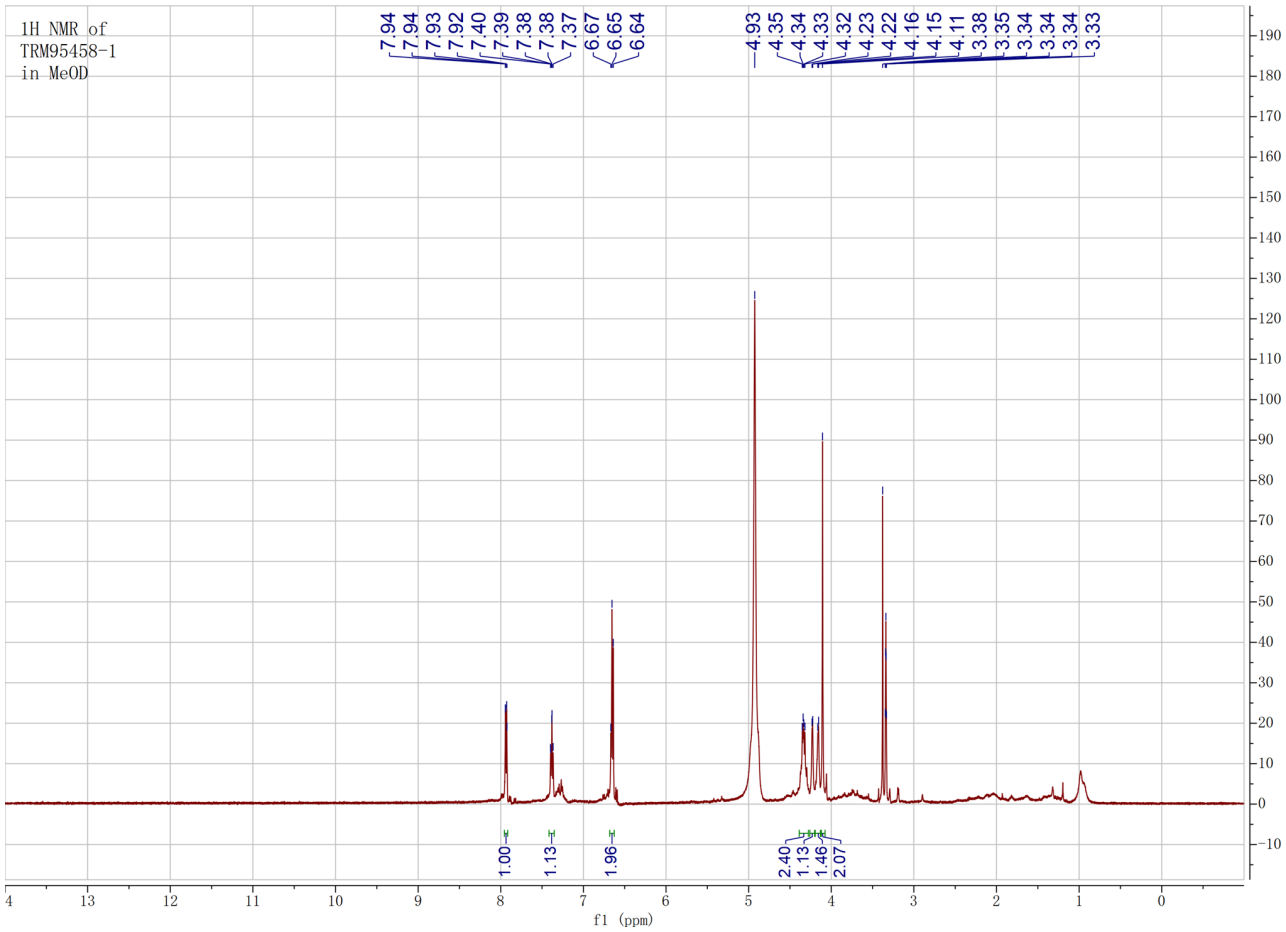
^1^H NMR of compound 1 in MeOD.

**Figure 6:**
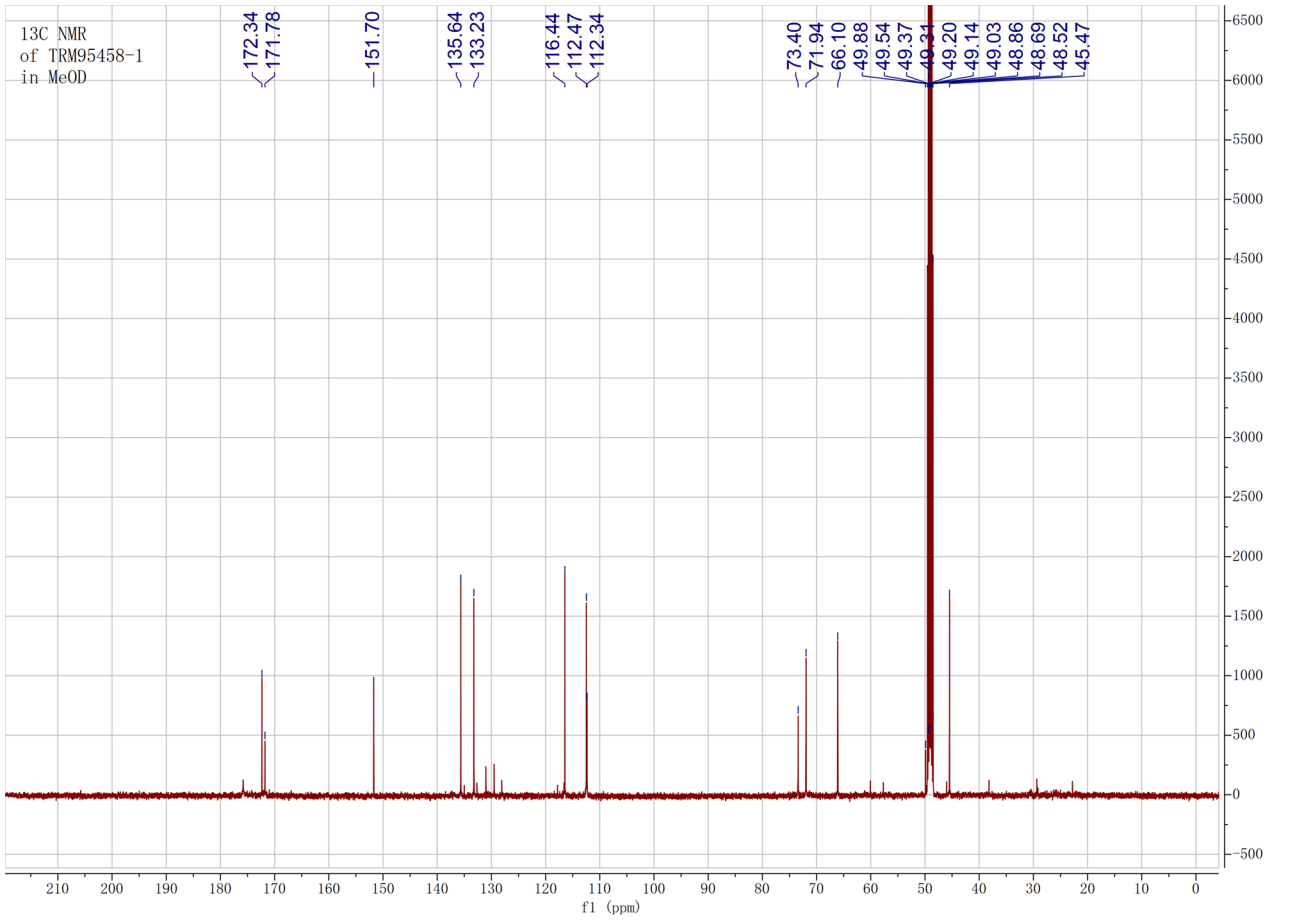
^13^C NMR of compound 1 in MeOD.

**Figure 7:**
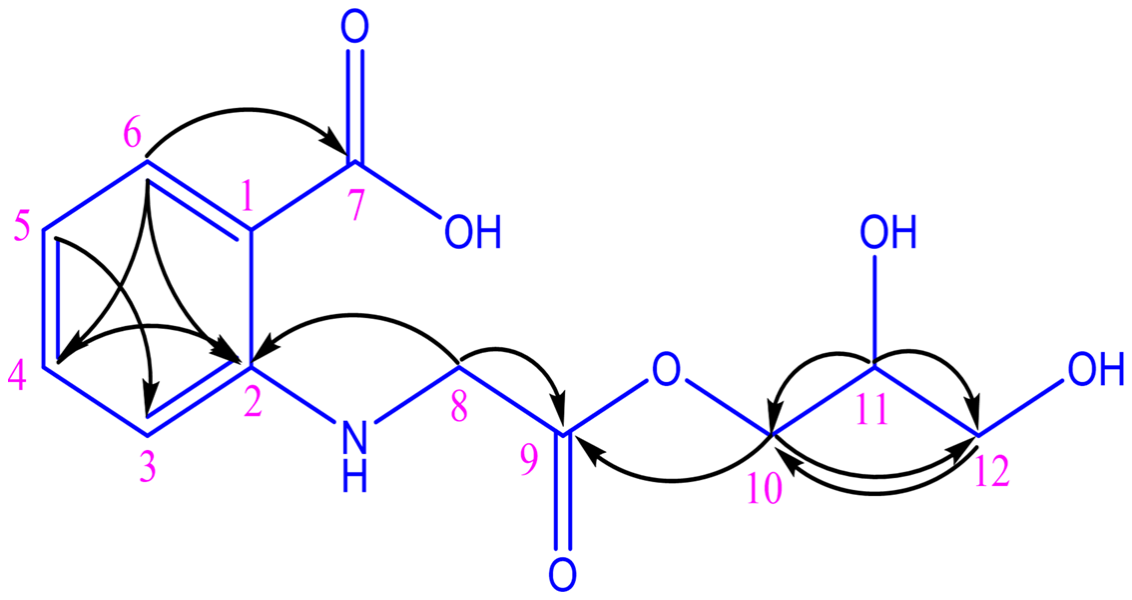
HMBC correlations of compound 1.

The organic extract of TRM 95458 cultured in a 20 L fermentation media was separated and purified to obtain compounds 1, 2, 3, and 4 (Figures 2, 3), among which compound 1 was a new compound. Compound 1 was obtained as a brown solid. Its HRESIMS (high-resolution electrospray ionization mass) data showed an (M + Na)^+^ ion peak at m/z 292.0793, indicating a molecular formula of C_12_H_15_NO_6_ (Figure 4). The ^1^H and ^13^C NMR spectra showed typical signals for the derivative of anthranilic acid (Figures 5, 6; Table 1). The high field area shows 7 proton signals according to the typical H signal. The peak at δ_H_ 4.11 (2H, s) is the methylene peak of C-8. The peak at δ_H_ 4.34 (2H, m) was the methylene peak of C-10. The peak at δ_H_ 4.23 (^1^H, m) was the peak of C-11. The peak at δ_H_ 4.16 (2H, m) was the methylene peak of C-12. The four H signals at 6 ~ 8 ppm were signals on the benzene ring. The ^13^C NMR spectrum as well as HSQC spectrum showed 12 carbon atoms, consisting of 3 secondary carbons, 5 tertiary carbons and 4 quaternary carbons with two carbonyl signals at δ_C_ 170 ~ 173 (Supplementary Figure S1). According to the HMBC spectrum, H-8 (δ_H_ 4.11) was associated with two quaternary carbon atoms (C-2, δ_C_ 151.70; C-9, δ_C_ 172.34), indicating glycine group connected with anthranilic acid. H-10 (δ_H_ 4.34) showed HMBC relationship to C-9 and C-12 (C-9, δ_C_ 172.34; C-12, δ_C_ 71.94), indicating glycerol group connected with glycine (Figure 7 and Supplementary Figure S2). Based on the 1D & 2D NMR (Supplementary Figure S3) spectroscopic analyses the new compound was ABAGG (compound **1**, 2-((2-(2,3-dihydroxypropoxy)-2-oxoethyl) amino) benzoic acid), together with three known compounds including 2-aminobenzoic acid (compound **2**), 2-((carboxymethyl) amino) benzoic acid (compound **3**), and 2-((2-methoxy-2-oxoethyl) amino) benzoic acid (compound **4**).

### Conversion from glycerol to ABAGG by TRM 95458

**Figure 8:**
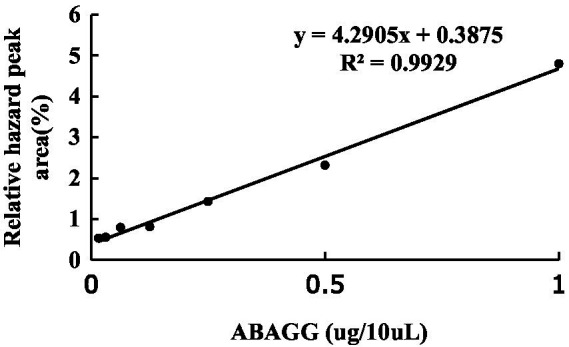
The standard curve of ABAGG by HPLC.

**Figure 9:**
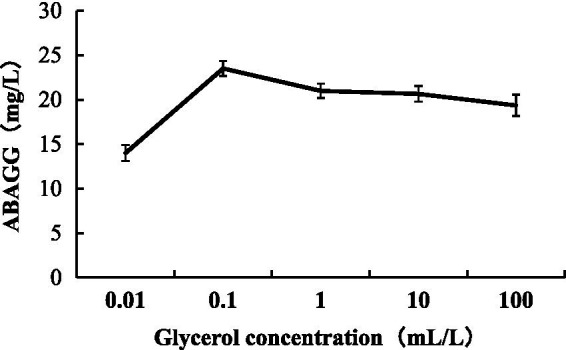
Production of ABAGG by TRM 95458 with different glycerol addition.

**Figure 10:**
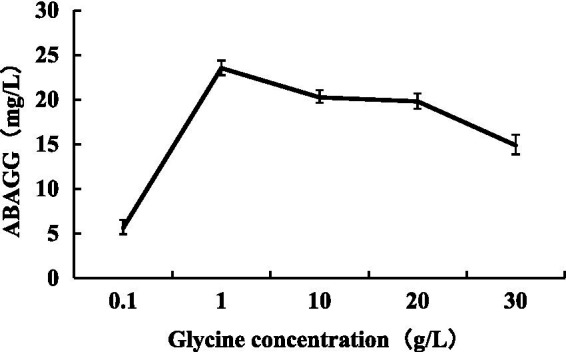
Production of ABAGG by TRM 95458 with different glycine addition.

The standard curve was determined by HPLC through the area normalisation method (Figure 8). The standard curve equation of ABAGG is *y* = 4.2905x + 0.3875, where x was the relative peak area, and *y* was the content of ABAGG. According to *R*^2^ = 0.9929, the linear relationship was good and was used to determine the content of ABAGG. Different contents of glycerol and glycine were added to the fermentation media resulted in accumulation of different amounts of ABAGG. The highest yield of ABAGG was obtained when 0.1 mL glycerol and 1 g glycine were added. Figure 9 showed the yield of ABAGG produced by *M. profundi* TRM 95458 with different glycerol additions. Figure 10 showed the yield of ABAGG produced by *M. profundi* TRM 95458 with different glycine addition.

**Table 1:** ^1^H NMR and ^13^C NMR data (125 MHz, MeOD) of 1.

Position	*δ*_C_	*δ*_H_ (*J* in Hz)
1	112.47	
2	151.70	
3	112.34	6.64 d (7.5)
4	135.64	7.38 t (7.5)
5	151.70	6.65 t (7.5)
6	133.23	7.94 d (7.5)
7	171.78	
8	45.47	4.11 2H, s
9	172.34	
10	66.10	4.23 2H, m
11	73.40	4.23 m
12	71.94	4.16 2H, m

### Salt stress regulation of the accumulation of ABAGG

**Figure 11:**
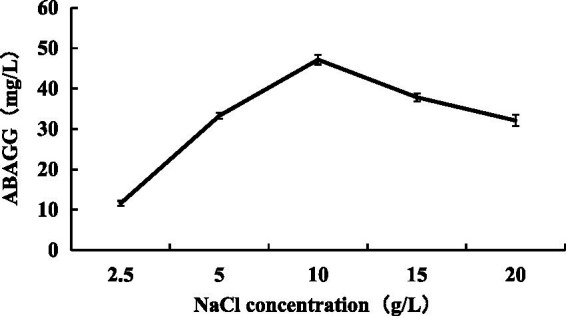
Relationship between salt stress and ABAGG accumulation in TRM 95458.

Changing NaCl concentration in the fermentation media varying from 2.5, 5, 10, 15 to 20 g/L, the yield of ABAGG in fermentation broth increased at first and then decreased with the increasing of NaCl concentration. When the NaCl content was 10 g/L, the yield of ABAGG was the highest (Figure 11). The results indicated that the salt tolerance of *M. profundi* TRM 95458 was improved by transforming glycerol and glycine and accumulating ABAGG in the cells under high salt concentration, indicating ABAGG as a new structure of an osmoregulatory-compatible substance.

### Screening of genes related to the conversion of glycerol and glycine

The genome size of strain TRM 95458 was 6.90 Mb, and the G + C content was 70%. Through the whole genome analysis of strain TRM 95458, the gene cluster contained salt tolerance-related genes, and the genes related to glutamine-related hydrolase, synthetase and aspartic acid synthase were screened (Table 2).

**Table 2:** Genes related to salt tolerance of strain TEM95458.

Seq ID	Function	Gene-name	Total
ctg_02190	Glutamine-hydrolyzing GMP synthase	guaA	1
ctg_03194	Glutamine amidotransferase	gatD	1
ctg_03398	Asparagine synthase (glutamine-hydrolyzing)	asnB	4
ctg_04519			
ctg_04667			
ctg_04771			
ctg_06205	Glutamine synthetase	glnA	3
ctg_03015			
ctg_02988			

## Discussion

The increasing global population and decreasing availability of cultivated land pose threats to sustainability due to the loss of irrigation water resources, soil salinization, and water and environmental pollution. Among these challenges, soil salinity is particularly devastating. Soil microbes play a crucial role in mitigating salt stress (Wang et al., 2020; Yukun et al., 2021). A significant number of published studies have focused on the isolation of salt-tolerant bacteria from soil rhizosphere microorganisms. Many actinomyces were isolated from the plant rhizosphere and were known to enhance plant growth and protect plant health (Liu et al., 2019). *Streptomyces* strain D2-8 was isolated from the rhizosphere of Phragmites, a plant that thrives in saline and alkaline environments. Strain D2-8 exhibited moderate tolerance to saline and alkaline stress, as it was able to grow in solutions containing 10% NaCl and 120 mM soda saline (Gao et al., 2022). The CZ-6 strain of antagonistic bacteria was isolated from the rhizosphere of wheat in saline soil. This strain has the ability to survive in a medium with a NaCl concentration of 10% and produces indole acetic acid (IAA) and 1-aminocyclopropane-1-carboxylic acid (ACC) deaminase. Due to these characteristics, CZ-6 shows potential as a biological control agent in saline soil (Zhou et al., 2021). In light of the significant potential of actinomycetes in mitigating salt stress conditions, this study focused on isolating and identifying *M. profundi* TRM 95458 from the chickpea rhizosphere soil in saline-alkali land in Xinjiang. The strain was found to possess the ability to convert glycerol and glycine into a novel osmotic compound called ABAGG. Under salt-stressed conditions, the majority of rhizospheric bacteria secrete osmo-protectants such as glutamate, trehalose, proline, proline betaine, and ectoine to maintain their cytoplasmic osmolarity (Kumar and Verma, 2018). These solutes, with their small molecular weight, are highly soluble. They play a crucial role in increasing intracellular water activity without disrupting normal cell metabolism (Kloska et al., 2022). The accumulation of these compatible solutes helps to balance the osmotic pressures inside and outside the cells, thereby alleviating the stress caused by high-salt environments. Additionally, the rapid synthesis and degradation of these solutes are advantageous for halophilic microorganisms in adapting to high osmotic pressures. Halophilic microorganisms can synthesize different compatible solutes according to the external conditions, such as duration of the osmotic stress, level of salinity, availability of substrates, and osmolytes in the surroundings. Beneficial microorganisms, such as *Azospirillum, Burkholderia, Arthrobacter, Bacillus, Pseudomonas,* and *Rhizobium*, have been found to contribute to the development of osmo-protectants. These osmo-protectants include proline, betaine, trehalose, glycine, phenols, and flavonoids (Hashem et al., 2019). *Streptomyces albidoflavus* OsiLf-2 is an endophytic actinomycete that exhibits moderate salt tolerance, with a tolerance of approximately 6% NaCl. This strain is capable of synthesizing important osmolytes such as proline, polysaccharides, and ectoine. Upon inoculation, the proline content in rice increased by 250.3% and 49.4%, respectively, under higher salinity. Similarly, the soluble sugar content in rice also showed an increase of 20.9% and 49.4%, respectively, under higher salinity (Niu et al., 2022). The three seaweeds used as soil amendments effectively mitigated the detrimental impact of salinity on canola plants. The seaweeds increased the levels of antioxidative compounds like phenols, flavonoids, anthocyanin, and osmo-protectants, including total carbohydrates and proline (Hashem et al., 2019). *Nocardiopsis* is an intriguing halophilic microorganism known for its ability to synthesize a diverse range of compatible solutes. These include ectoine, hydroxyectoine, trehalose, glutamate, and β-glutamate, among others. Halotolerant The ectABC from *Nocardiopsis gilva* YIM 90087 T was activated under the salt stress (Han et al., 2018). The production of hydroxyectoine was observed in six bacterial isolates, namely *Nesterenkonia xinjiangensis, Halobacillus* sp.*, Halomonas neptunia, Thalassobacillus devorans, Nesterenkonia sp.,* and *Bacillus agaradhaerens*. The production was found to be dependent on NaCl concentration and temperature. Additionally, the study identified these bacterial isolates as novel producers of ectoine or hydroxyectoine (Orhan and Ceyran, 2023). All osmolytes share the common characteristic of reducing the osmotic potential in the cytosolic compartment at higher concentrations, without impeding metabolic reactions (Lou et al., 2018). Certain stress conditions are employed during the production of specific metabolites. *Cyanobacteria* have been observed to adapt to changing environmental conditions by adjusting the concentration of metabolites (Yadav et al., 2022). It is important to note that micro-based products have the potential to be more effective than microbial strains for different crop varieties and under various environmental conditions (Kumawat et al., 2020). The concentration of NaCl in the fermentation media had a clear impact on the production of ABAGG, indicating that salt stress resulted in the accumulation of ABAGG. Together, these results suggest that ABAGG and *M. profundi* TRM 95458 could potentially play a role in enhancing the survival and adaptation of plants in saline environments.

## Conclusion

In summary, *M. profundi* TRM 95458, a strain capable of converting glycerol and glycine, was isolated and identified from rhizosphere of chickpea. A new osmotic compound (ABAGG) was identified from the fermentation broth of *M. profundi* TRM 95458 by 1D & 2D NMR and HRMS data. The accumulation of ABAGG varied depending on the amount of glycerol and glycine added to the fermentation media. The concentration of NaCl in the fermentation media obviously affected the production of ABAGG, suggesting that salt stress led to the accumulation of ABAGG. ABAGG and *M. profundi* TRM 95458 might be implicated for improving the survival and adaptation of plants in saline environments.

## Data availability statement

The datasets presented in this study can be found in online repositories. The names of the repository/repositories and accession number(s) can be found at: NCBI – PRJNA994917, SAMN36452542, OR269619.

## Author contributions

C-xW: generated ideas, obtained funding, directed the work, chemical investigation, and reviewed the manuscript. DL: performed the experiments, collected, analyzed, and interpreted the data, prepared, and edited the manuscript. H-lS: isolated and identificated the strain of TRM 95458. LW: performed part of the experiments, analyzed, and interpreted the data. All authors contributed to the article and approved the submitted version.

## Funding

This study was supported by Bingtuan Science and Technology Program (2021BB007) and Tarim University Principal’s Fund Project (TDZKKY202201).

## Conflict of interest

The authors declare that the research was conducted in the absence of any commercial or financial relationships that could be construed as a potential conflict of interest.

## Publisher’s note

All claims expressed in this article are solely those of the authors and do not necessarily represent those of their affiliated organizations, or those of the publisher, the editors and the reviewers. Any product that may be evaluated in this article, or claim that may be made by its manufacturer, is not guaranteed or endorsed by the publisher.
