# Supplementary figures and images for: Micromonospora profundi TRM 95458 converts glycerol to a new osmotic compound
Source: Front Microbiol. 2023 Sep 7;14:1236906. doi: 10.3389/fmicb.2023.1236906 (PMC10513789; doi:10.3389/fmicb.2023.1236906)

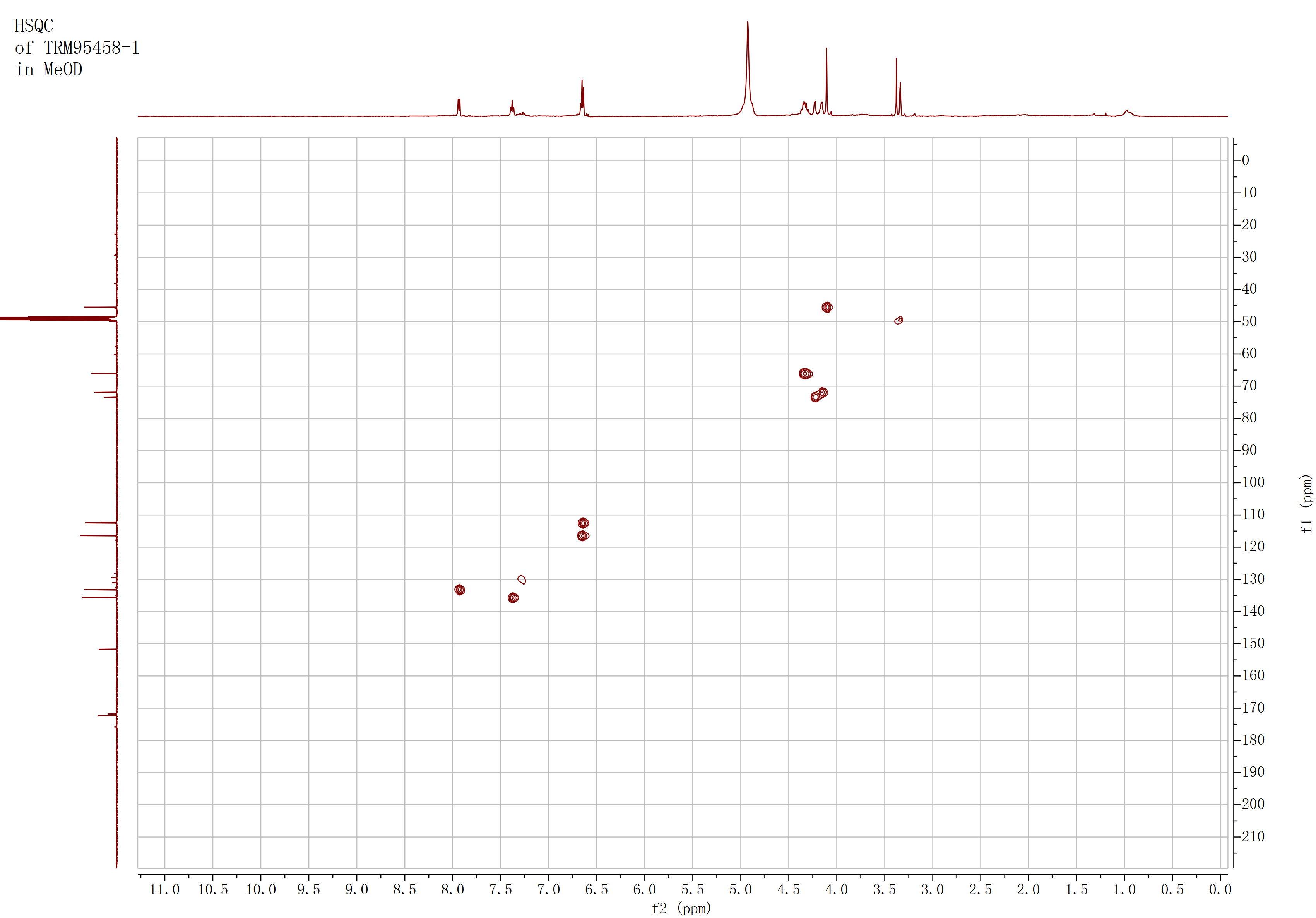

Supplement: Supplementary file 1 [file Image_1.tiff]

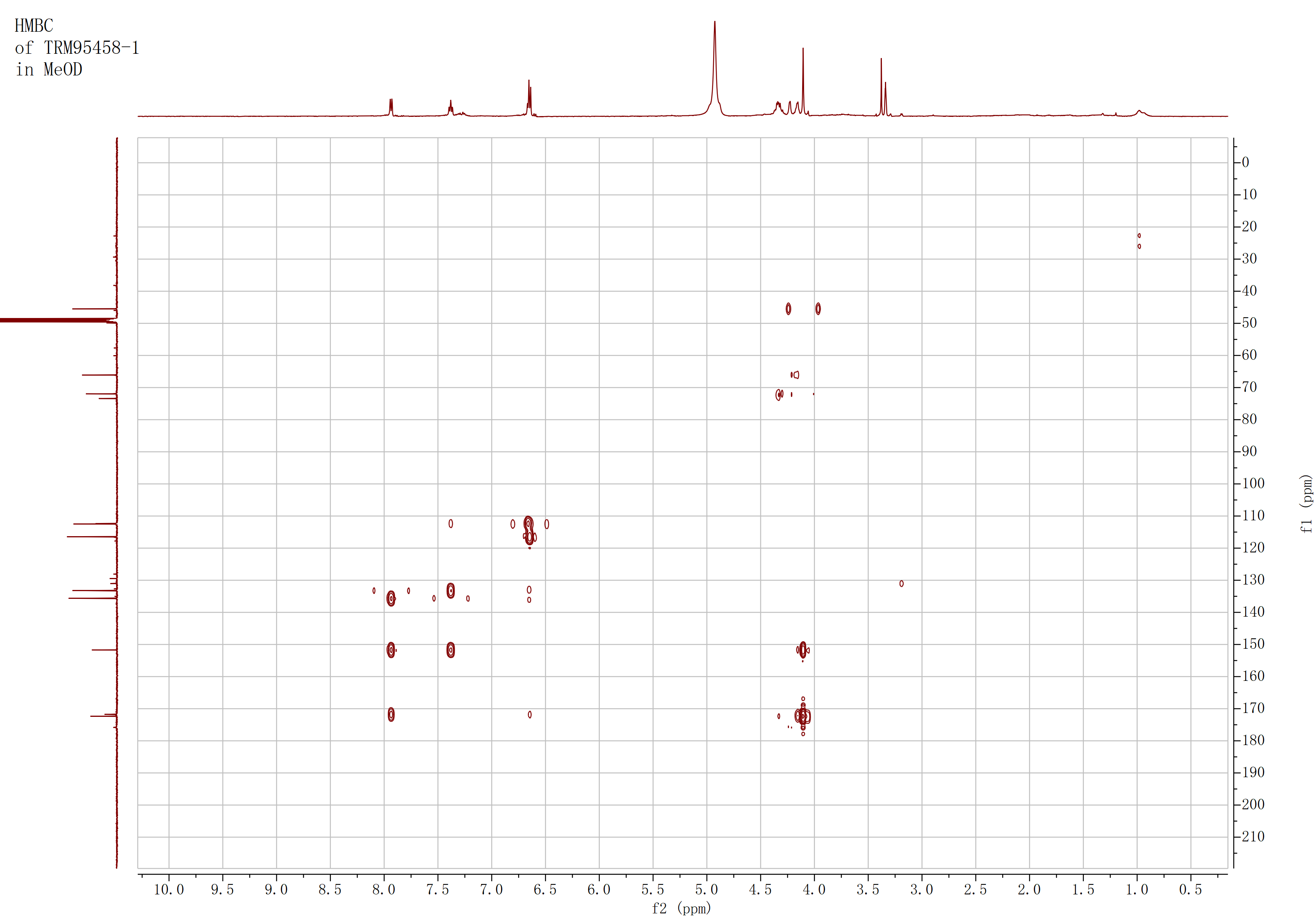

Supplement: Supplementary file 2 [file Image_2.tiff]

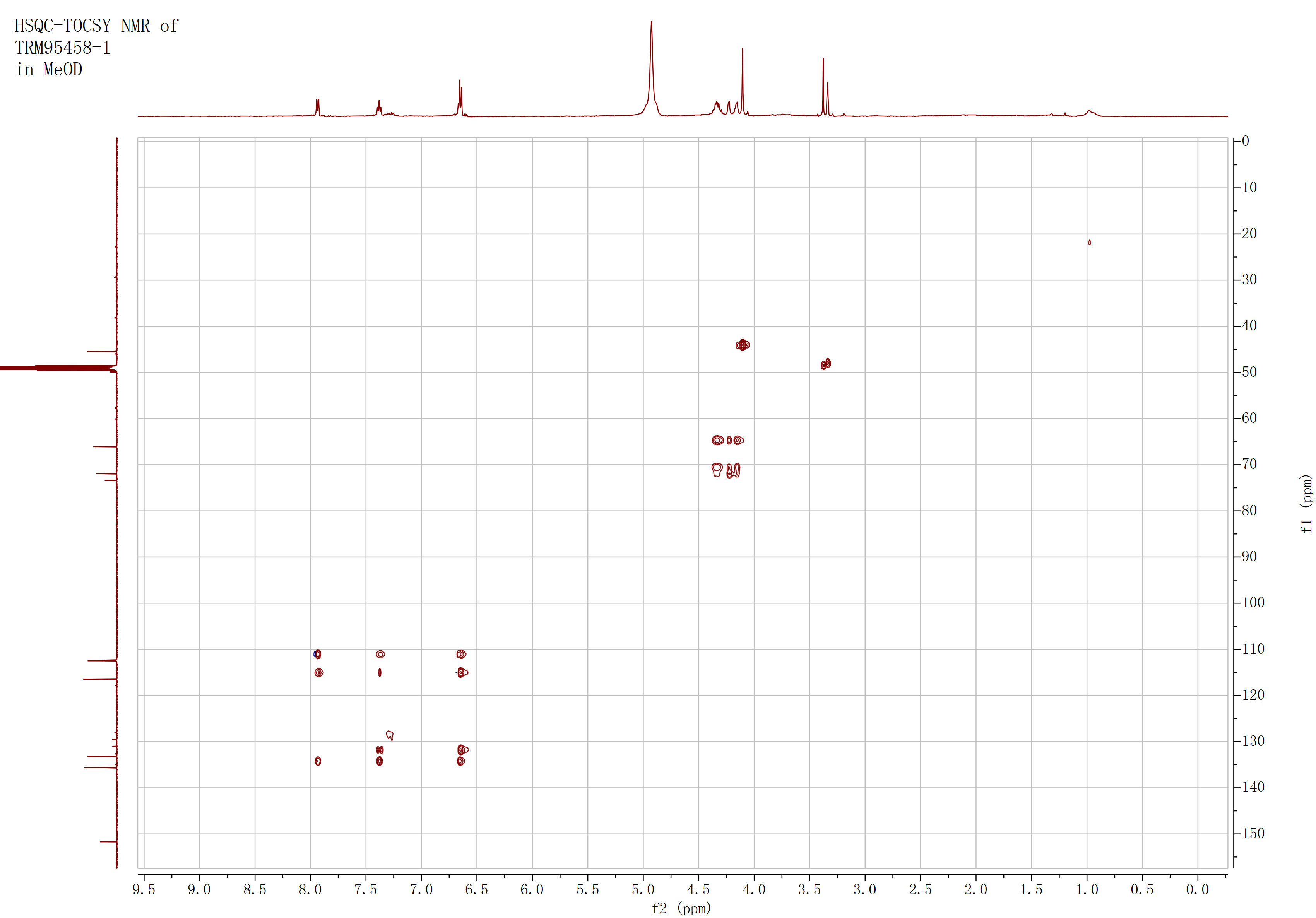

Supplement: Supplementary file 3 [file Image_3.tiff]
